# Dietary Capsiate-Producing Chili Pepper Promotes Somatic and Femoral Growth and Modulates Intestinal Immunometabolic Responses in Mice

**DOI:** 10.3390/molecules31101679

**Published:** 2026-05-15

**Authors:** Diana Vanesa Gutiérrez-Chávez, Estefanía Arellano-Ordoñez, Ana Angélica Feregrino-Pérez, Juan Fernando García-Trejo, Diana Catalina Castro-Rodríguez, Omar Granados-Portillo, Abigail García-Morales, Ramón Gerardo Guevara-González, Consuelo Lomas-Soria

**Affiliations:** 1 Biosystems Engineering Group, Center of Applied Research in Biosystems (CARB-CIAB), School of Engineering Campus Amazcala, Autonomous University of Queretaro, Queretaro 76260, Mexico; vanegcz@gmail.com (D.V.G.-C.);; 2 Academic Group of Basic and Applied Engineering, Faculty of Engineering, Autonomous University of Queretaro, Queretaro 76260, Mexico; 3 Research and Postgraduate Division, School of Engineering, Autonomous University of Queretaro, Campus Amazcala, Carretera a Chichimequillas Km 1 s/n, Amazcala, El Marques, Queretaro 76265, Mexico; 4 Department of Immuno-Biochemistry, Instituto Nacional de Perinatología, Mexico City 11000, Mexico; 5 Department of Nutritional Physiology, Instituto Nacional de Ciencias Médicas y Nutrición Salvador Zubirán, Mexico City 14080, Mexico; 6 Doctorate in Biological and Health Sciences, Metropolitan Autonomous University, Mexico City 04960, Mexico; 7 Reproductive Biology Department, Instituto Nacional de Ciencias Médicas y Nutrición Salvador Zubirán, Mexico City 14080, Mexico; 8 Center for Research on Aging (CIE-CINVESTAV Sur), Mexico City 14330, Mexico

**Keywords:** Capsiate, *Capsicum annuum*, TRPV1, immunometabolism, growth development, C57BL/6J mice, functional foods

## Abstract

Capsaicin has been investigated as a phytogenic feed additive in animal production due to reported growth-promoting and immunomodulatory properties; however, its pungency limits practical application. Capsiate, a naturally occurring non-pungent capsaicin analog present in specific *Capsicum annuum* accessions, conserves many of its bioactive properties without inducing sensory irritation and has not been studied as a potential growth-promoting alternative. The present study evaluated whether dietary exposure to a capsiate-producing chili pepper influences growth and assessed associated intestinal responses using a murine model. A capsiate-producing *Capsicum annuum* accession (509-45-1) was characterized and incorporated into experimental diets providing 30 or 50 mg/kg capsiate to male C57BL/6J mice for 12 weeks. The dietary intervention was associated with dose-dependent increases in body weight and longitudinal femoral growth without altering body composition. Femoral elongation was accompanied by increased growth plate area and higher osteocyte number and area. At the intestinal level, the intervention was associated with downregulation of colonic transient receptor potential vanilloid 1 (*TRPV1*) gene expression, modulation of redox-associated responses, including catalase (*CAT*) and superoxide dismutase (*SOD*) expression, and differential modulation of innate immune signaling, including upregulation of Toll-like receptor 2 (*TLR2*) and downregulation of Toll-like receptor 4 (*TLR4*), together with reduced interleukin-6 (*IL-6*) and tumor necrosis factor alpha (*TNF-α*) expression. Collectively, these findings indicate that dietary supplementation with a capsiate-producing chili is associated with increased somatic growth and enhanced femoral development in mice, accompanied by intestinal transcriptional changes consistent with immunometabolic responses, while preserving body composition.

## 1. Introduction

Antimicrobials have been widely used in livestock production for therapeutic, prophylactic, and metaphylactic purposes, as well as to promote growth and improve feed efficiency [1]. Their incorporation as feed additives has contributed to improved growth performance, feed efficiency, and overall animal health [2]. However, concerns regarding antimicrobial resistance associated with their misuse have prompted the search for natural alternatives to antibiotic growth promoters capable of providing comparable multifactorial benefits in sustainable animal production [3]. Among these alternatives, phytogenic additives have gained attention due to their capacity to influence growth performance, immune balance, oxidative status, and intestinal function across animal species [4].

Animal growth is strongly influenced by intestinal physiology and the gut environment. Increasing evidence indicates that host digestion operates in conjunction with microbial metabolism, generating bioactive metabolites such as short-chain fatty acids (SCFAs) that regulate barrier function, immune balance, and systemic metabolism [5]. In parallel, the intestinal redox environment contributes to shaping mucosal immune responses and inflammatory signaling [6]. Together, these processes position the gut as a key interface linking nutrient utilization with systemic growth regulation [7]. Consequently, phytogenic additives that modulate intestinal physiology and host–microbiome interactions may influence metabolic and inflammatory pathways involved in growth and tissue development [8]. However, the integrative mechanisms connecting intestinal modulation with systemic growth outcomes remain incompletely understood, highlighting the need for controlled studies evaluating intestinal and growth-related physiological responses.

Among phytogenic bioactives, compounds derived from *Capsicum* species have attracted particular interest in livestock production due to their metabolic and immunomodulatory properties [9]. Chili peppers (*Capsicum* spp.) are rich sources of bioactive molecules, particularly capsaicinoids and their non-pungent analogs, capsinoids [10]. Capsaicin, the principal pungent compound, has been reported to modulate growth performance, inflammatory responses, oxidative balance, and microbial dynamics in livestock and experimental models [9,10]. However, capsaicin exhibits strong pungency, limited water dispersibility, and reduced bioaccessibility, which restrict its broader application [11].

Capsiate, a naturally occurring non-pungent analog, shares similar chemical features and biological properties while lacking sensory irritation [12]. Previous studies have associated capsiate intake with enhanced energy metabolism [13], improved intestinal function [14,15] and protection against oxidative and inflammatory stress [16]. Nevertheless, whether capsiate enhances growth performance similarly to capsaicin, as well as whether such effects are associated with intestinal and systemic physiological responses, remains unexplored.

*Capsicum annuum L.* accession 509-45-1 has been identified as a natural producer of capsiate [17], providing a non-pungent chili source suitable for dietary exposure without sensory limitations. Based on this background, the present study aimed to characterize this capsiate-producing accession and to evaluate whether its dietary administration promotes growth in a murine model. To better understand growth outcomes, body weight, body composition, and femoral length were assessed as indicators of somatic development. Particular emphasis was placed on intestinal redox and inflammatory responses as potential mediators linking colonic functional adaptations with growth. By integrating phenotypic, molecular, and tissue-level analyses, this study explores the mechanistic basis of capsiate-induced somatic and skeletal growth and provides insight into gut-mediated regulation of bone development.

## 2. Results

### 2.1. Chemical Composition of Capsiate-Producing Chili (C. annuum L.)

The fruits of *Capsicum* spp. exhibit extensive morphological and chemical variability influenced not only by genetic factors but also by environmental and crop management conditions [18]. The chemical composition of capsiate-producing chili (*Capsicum annuum* L. accession 509-45-1) is summarized in Table 1. Capsaicin and dihydrocapsaicin were not detected, as expected, due to alterations in the capsaicinoid biosynthetic pathway present in this variety. Specifically, the conversion of vanillin into vanillyl alcohol via cinnamyl alcohol dehydrogenase (CAD) favors capsiate synthesis [19], resulting in capsiate as the only capsaicinoid identified.

Additionally, this variety contains significant levels of phenolic compounds and flavonoids, which contribute to its antioxidant capacity. This was primarily reflected by radical scavenging activity (RSA), determined using the ABTS assay (2,2′-azinobis(3-ethylbenzothiazoline-6-sulfonic acid)) and expressed as percentage (%) inhibition (62.48 ± 1.08%). In contrast, DPPH radical scavenging activity (2,2-diphenyl-1-picrylhydrazyl) was considerably lower (16.11 ± 1.25%), which may be attributed to differences in solvent compatibility and reaction mechanisms, as ABTS is suitable for evaluating both hydrophilic and lipophilic antioxidant systems, whereas DPPH is more limited to hydrophobic environments. Protein content was detected in low amounts, while dietary fiber was abundant, particularly insoluble fiber (Table 1).

### 2.2. Effects of Chili Supplementation on Growth Performance and Metabolic Parameters

#### 2.2.1. Weight Gain

**Figure 1:**
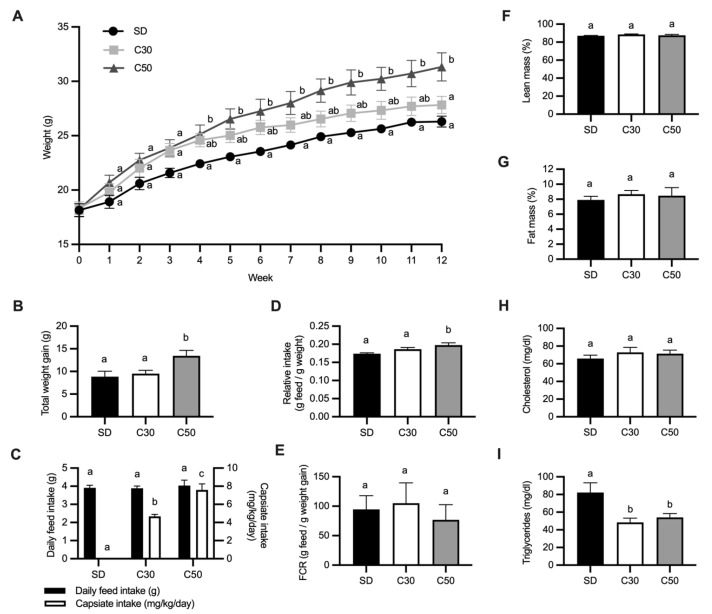
Effect of *C. annuum* on weight, food intake, body composition and serum lipids in mice. (**A**) Weekly weight gain, (**B**) total weight gain in twelve weeks, (**C**) average daily food intake and estimated capsiate intake, (**D**) average daily relative intake per mouse by treatment, (**E**) feed conversion ratio, (**F**) lean mass percentage, determined by magnetic resonance imaging, (**G**) fat mass percentage, determined by magnetic resonance imaging, (**H**) total cholesterol content in serum, (**I**) triglyceride content in serum. Data not sharing a letter are statistically different. Data are expressed as means ± SEM. *n* = 7 mice per group.

As shown in Figure 1A, capsiate-producing chili exerted a dose-dependent effect on mouse body weight. From weeks four to twelve, mice in the C50 group consistently exhibited greater weight gain compared to the SD and C30 groups, indicating a growth-promoting effect at 50 mg capsiate/kg feed. In contrast, the C30 group followed a weight gain pattern comparable to that of the SD group throughout most of the experiment, with only slight divergence from the C50 group in the final week. By the end of the study, mice in the C50 group gained a total of 13.45 g, significantly higher than that gained by the SD group (8.86 g), corresponding to a 52% increase in body weight gain (Figure 1B).

#### 2.2.2. Effects on Feed Intake and Feed Conversion Ratio (FCR)

Feed intake (FI) and relative intake (RI; normalized to body weight) were evaluated to assess whether differences in weight gain were associated with changes in food consumption. Based on FI, the estimated capsiate intake was 4.67 and 7.59 mg/kg/day for the C30 and C50 groups, respectively (Figure 1C). No significant differences in average daily FI were observed among treatments.

Although RI was significantly higher in the C50 group compared to the SD group (C50: 0.197 ± 0.02 vs. SD: 0.174 ± 0.01), the magnitude of this increase was modest (approximately 13%; Figure 1D), particularly when contrasted with the substantially greater increase in body weight (~52%). This suggests that the enhanced weight gain observed in the C50 group cannot be explained solely by increased feed intake relative to body weight. Consistently, no significant differences were detected in the feed conversion ratio (FCR) among groups (Figure 1E).

#### 2.2.3. Body Composition Measurement by Magnetic Resonance Imaging (MRI)

Body composition analysis by magnetic resonance imaging (MRI) revealed no significant differences in either lean mass (Figure 1F) or fat mass (Figure 1G) among groups. These findings indicate that, despite the greater weight gain observed in the C50 group, this effect was not associated with increased adiposity.

#### 2.2.4. Hepatic, Adipose Tissue, and Serum Changes

Biochemical analysis showed no significant differences in serum cholesterol levels between groups (Figure 1H). However, mice receiving chili supplementation exhibited a significant reduction in fasting serum triglyceride levels compared to the SD group (Figure 1I).

Additionally, as shown in Table 2, dietary supplementation with capsiate-producing chili at 30 and 50 mg/kg did not affect relative liver weight or fat distribution across different anatomical depots following dissection.

### 2.3. Histological Changes in Liver, Jejunum, and Femur

**Figure 2:**
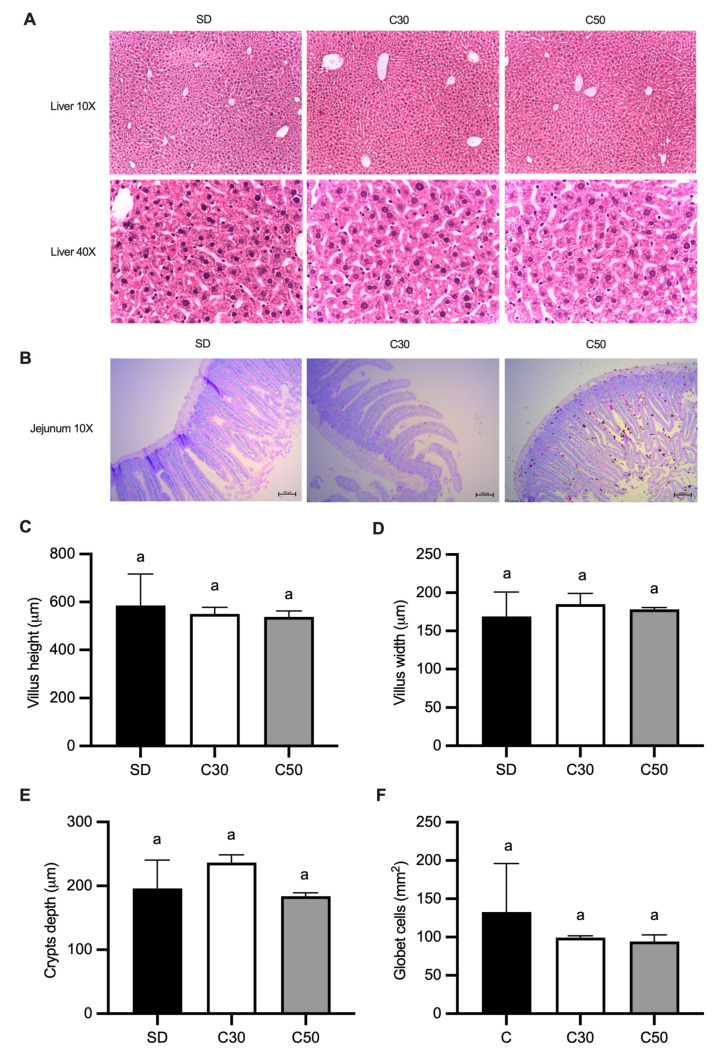
Effect of *C. annuum* on liver and intestinal mice morphology. (**A**) Liver histology (**B**) jejunum histology, (**C**) villus height, (**D**) villus width, (**E**) crypt depth and (**F**) goblet cells. Data are expressed as means ± SEM. Data not sharing a letter are statistically different; *p* < 0.05.

Histological evaluation using hematoxylin and eosin staining (H&E) showed no morphological alterations in liver tissue across experimental groups (Figure 2A). Similarly, no histopathological changes were observed in the jejunum (Figure 2B). Morphometric analysis indicated that dietary supplementation with capsiate-producing chili at 30 and 50 mg/kg feed did not affect villus height or width, crypt depth, or goblet cell number (Figure 2C–F).

**Figure 3:**
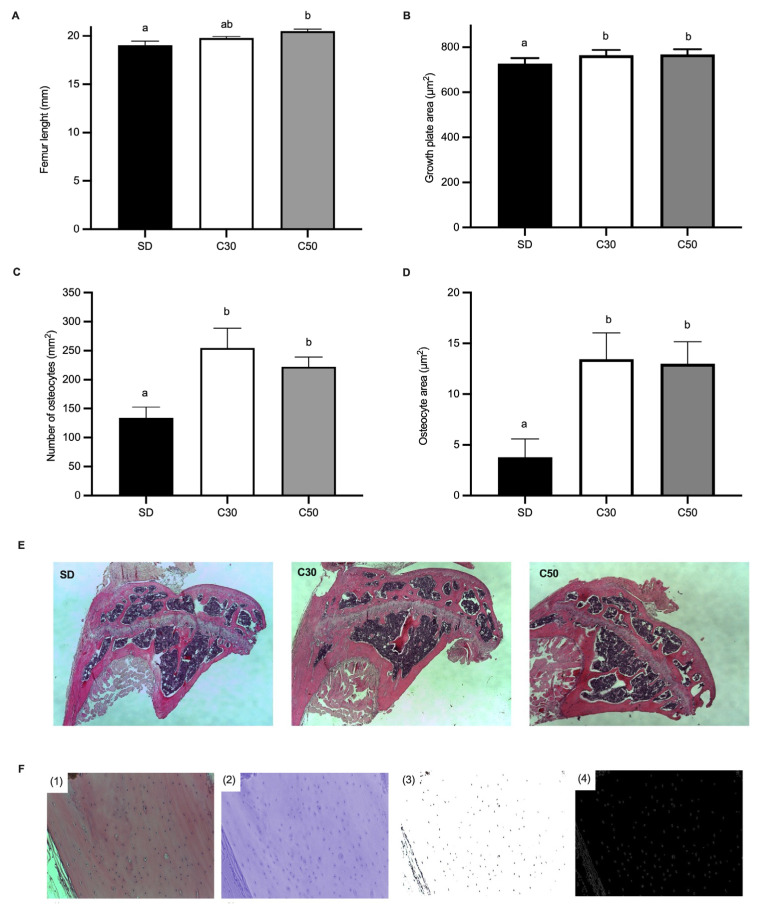
Effect of *C. annuum* on mice femur morphology in mice. (**A**) Femur length, (**B**) growth line area, (**C**) number of osteocytes per area, (**D**) average osteocyte size, (**E**) representative image of the femoral growth plate from an individual from each treatment, (**F**) image analysis workflow for osteocyte quantification and morphometric analysis. The workflow includes: (**F1**) acquisition of the original femoral tissue image stained with H&E; (**F2**) color normalization and contrast enhancement; (**F3**) thresholding and binarization to isolate osteocyte lacunae; and (**F4**) contour and edge detection for automated osteocyte counting and area determination. Data are expressed as mean ± SEM. Data not sharing a letter are significantly different (*p* < 0.05).

As shown in Figure 3, femur length was greater in the C50 group compared with the SD group (Figure 3A). Histological analysis further revealed an increased growth plate area in mice supplemented with chili (Figure 3B), accompanied by a higher number of osteocytes and increased osteocyte area (Figure 3C,D). Representative sections of the femoral growth plate are presented in Figure 3E, illustrating structural differences among groups.

To support these findings, an image analysis workflow was implemented for osteocyte identification and morphometric quantification (Figure 3F). Briefly, femoral sections stained with H&E were subjected to color normalization and contrast enhancement, followed by thresholding and binarization to isolate osteocyte lacunae. Contour and edge detection were then applied to automated osteocyte counting and area determination. This analysis confirmed that mice receiving capsiate-producing chili at 30 and 50 mg/kg exhibited larger osteocytes compared to the SD group.

### 2.4. Transcriptional Changes Assessed by RT-qPCR

Gene expression analysis by reverse transcription quantitative PCR (RT-qPCR) was performed to evaluate antioxidant, inflammatory, receptor, and intestinal barrier markers in jejunal and colonic tissues.

**Figure 4:**
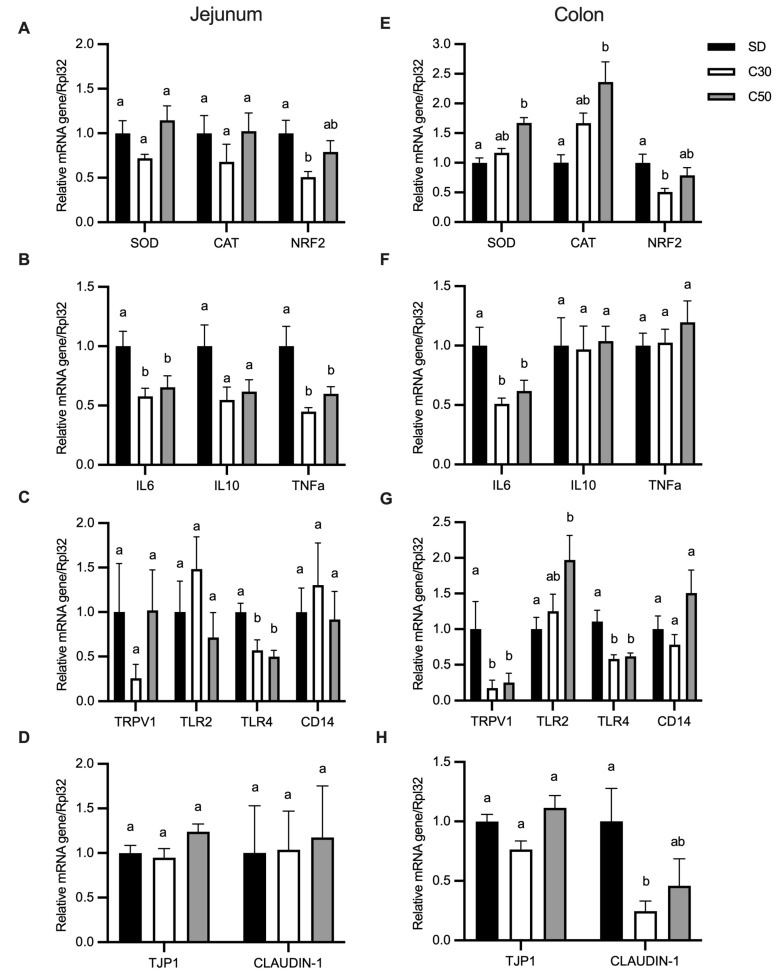
Effect of *C. annuum* on gene expression of antioxidant, inflammatory, receptor, and intestinal barrier markers in mouse jejunum and colon. Jejunal expression of genes related to (**A**) antioxidant activity, (**B**) inflammatory response, (**C**) receptors, and (**D**) intestinal barrier proteins. Colonic expression of genes related to (**E**) antioxidant activity, (**F**) inflammatory response, (**G**) receptors, and (**H**) intestinal barrier proteins. Data are expressed as mean ± SEM. Values not sharing a letter are significantly different (*p* < 0.05).

In the jejunum, the expression levels of superoxide dismutase (*SOD*) and catalase (*CAT*) showed no significant differences among the groups. In contrast, nuclear factor erythroid 2-related factor 2 (*NRF2*) expression was significantly decreased in the C30 treatment compared to SD, while remaining unchanged in the C50 group (Figure 4A). In the colon, *SOD* and *CAT* expression were significantly upregulated in the C50 group compared to SD, whereas *NRF2* expression was downregulated in the C30 group (Figure 4E), suggesting segment-specific modulation of antioxidant responses.

Analysis of inflammatory markers revealed that interleukin-6 (*IL-6*) expression was significantly reduced in the jejunum of both C30 and C50 groups (Figure 4B). Interleukin-10 (*IL-10*) showed no significant differences, although a decreasing trend was observed in C30 compared to SD (α = 0.0681). Tumor necrosis factor alpha (*TNFα*) was also significantly reduced in the jejunum of both treatment groups (Figure 4B). In the colon, only *IL-6* expression was decreased in the capsiate-treated groups, while *IL-10* and *TNFα* remained unchanged (Figure 4F), indicating a region-dependent modulation of inflammatory signaling.

The mRNA expression receptors exhibited differential patterns along the intestinal tract. In the jejunum, transient receptor potential vanilloid 1 (*TRPV1*) and toll-like receptor 2 (*TLR2*) expression remained unchanged, whereas toll-like receptor 4 (*TLR4*) was downregulated in both C30 and C50 groups compared to SD. Cluster of differentiation 14 (*CD14*) expression was not affected (Figure 4C). In the colon, *TRPV1* and *TLR4* were downregulated in both treatment groups, while *TLR2* was upregulated in C50, and *CD14* remained unchanged (Figure 4G). These results suggest site- and receptor-specific modulation by capsiate.

Regarding intestinal barrier-related genes, tight junction protein 1 (*TJP1*) expression remained unchanged in both jejunum and colon (Figure 4D,H). In contrast, *claudin-1* expression in the colon was significantly reduced in the C30 group compared to SD (Figure 4H), indicating a selective effect on tight junction components.

### 2.5. Fecal SCFA Profile

**Figure 5:**
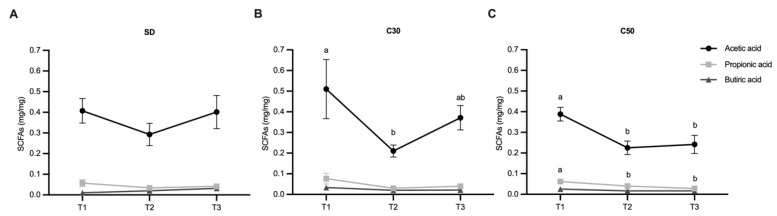
Fecal SCFA content. (**A**) SCFA quantification in mice fed a standard diet (SD) at one week (T1), eight weeks (T2), and twelve weeks (T3); (**B**) SCFA levels in mice supplemented with 30 mg/kg capsiate; and (**C**) SCFA levels in mice supplemented with 50 mg/kg capsiate at the same time points. Data are expressed as mean ± SEM. Values not sharing a letter are significantly different (*p* < 0.05).

Fecal short-chain fatty acids (SCFAs) were quantified at different time points throughout the experimental period. A temporal modulation of acetic acid levels was observed in chili-supplemented groups (Figure 5B,C). In the C30 group, fecal acetic acid content decreased significantly at T2 compared to T1. In the C50 group, acetic acid levels were significantly reduced at both T2 and T3 relative to T1.

In addition, the C50 group exhibited a significant reduction in propionic acid from T1 to T2, which remained low at T3 (Figure 5C). In contrast, no further changes were observed in propionic or butyric acid levels in the SD and C30 groups across the evaluated time points.

## 3. Discussion

Variation in the chemical composition of *Capsicum* species represents an important physiological trait for exploring genotype-dependent bioactivity and regulatory potential [18]. Accession 509-45-1 displayed a distinctive capsinoid profile, naturally producing 0.55 mg/g FW of capsiate, which doubled after elicitation with H_2_O_2_ [17]. Dried samples reached 4.13 ± 0.16 mg/g DW, exceeding previous reports and surpassing CH-19 Sweet under standard conditions [20]. This accession was also rich in phenolics (34.99 ± 2.02 mg GAE/g DW) and flavonoids (27.37 ± 1.55 mg RE/g DW), with strong antioxidant activity (DPPH 16.11 ± 1.25%; ABTS 62.48 ± 1.08%), supporting its relevance as a bioactive dietary source. Protein and fiber contents were comparable to or higher than those reported for typical *C. annuum* cultivars [18], further highlighting its nutritional value.

The biological activity of this accession is likely driven by its chemical composition, including its capsinoid profile and other bioactive constituents such as phenolics, flavonoids, and dietary fiber. Capsiate differs structurally from capsaicin, which may influence its bioavailability, metabolic stability, and interaction with molecular targets such as TRPV1 [9], while phenolics and flavonoids may further contribute to the antioxidant and immunomodulatory effects.

Dietary supplementation with chili providing 50 mg capsiate/kg feed (C50) did not alter absolute feed intake; however, relative intake (RI) was modestly higher (~13%) and feed conversion ratio (FCR) remained unchanged. Under these conditions, C50 was associated with increased body weight over the intervention period, whereas the lower dose (30 mg capsiate/kg feed; C30) did not result in measurable growth effects. Importantly, this increase in body weight occurred without detectable changes in fat and lean mass, adipose tissue distribution, or liver weight.

Alongside these outcomes, femoral length and growth plate area were significantly increased in chili-supplemented mice, together with higher osteocyte number and size. These findings indicate an association between consumption of a capsiate-producing chili pepper and enhanced femoral growth parameters under the experimental conditions evaluated. However, the underlying mechanisms remain to be elucidated.

Chili supplementation also modulated lipid metabolism, as evidenced by reduced serum triglyceride concentrations without alterations in total cholesterol. This pattern is consistent with previous reports describing associations between capsinoids and modulation of lipid metabolism [21]. The absence of detectable changes in body composition likely reflects the effective daily dose achieved in this study (7.59 mg/kg/day estimated by average FI), which was slightly below thresholds previously associated with reductions in fat mass and increases in lean mass (~10 mg/kg/day) [22,23].

Intestinal morphology plays a critical role in nutrient absorption and gastrointestinal function [24]. At the intestinal level, supplementation with capsiate-producing chili pepper did not induce detectable changes in jejunal morphology, suggesting that the functional and molecular responses observed in this study occurred in the absence of structural remodeling of the mucosa. This differs from reports describing structural intestinal alterations after capsaicin exposure [9].

Instead, the intervention induced dose-dependent modulation of transcriptional responses across intestinal genes involved in sensory signaling, redox regulation, barrier function, and immune pathways. Reduced expression of *TRPV1* and *TLR4*, together with lower *IL-6* levels, is consistent with attenuation of sensory–innate immune signaling and inflammatory transcriptional tone [25]. Reduced intestinal inflammatory activity has been associated with lower energetic costs linked to sustained immune activation, potentially favoring nutrient allocation toward growth-related processes [26].

*TRPV1*, a member of the transient receptor potential vanilloid family, is widely expressed in intestinal epithelial and sensory neurons, where it participates in gastrointestinal physiology and neuroimmune pathways involved in the regulation of intestinal inflammatory responses [27]. In the present study, this reduction in *TRPV1* expression following dietary exposure to capsiate-producing chili may reflect adaptive transcriptional responses consistent with mechanisms previously associated with sustained exposure to vanilloid compounds [28].

In parallel, modulation of *NRF2* together with changes in *SOD* and *CAT* expression suggests adjustments of redox-associated gene expression [29]. Tight junction-related genes also showed dose-dependent transcriptional regulation, with *Claudin-1* decreasing at the lower dose and normalizing at the higher dose, while *TJP1* remained unchanged, suggesting fine-tuned regulation of epithelial barrier-related gene expression [30]. Together with the absence of detectable morphological alterations in histological analyses, these findings support transcriptional regulation of intestinal function-related genes without evidence of structural compromise.

Conversely, *TLR2* gene expression increased in the colon at higher capsiate doses, which may reflect adaptive mucosal immune signaling under a reduced inflammatory environment. *TLR2* recognizes microbial ligands, primarily derived from Gram-positive commensal bacteria, and contributes to maintaining epithelial barrier integrity and host–microbe communication in the intestine [31]. Moreover, *TLR2* activation has been associated with enhanced tight-junction expression and barrier stabilization in response to beneficial microbial stimuli [32]. Thus, increased *TLR2* expression, together with reduced *TLR4* and pro-inflammatory cytokines, may reflect enhanced mucosal surveillance and barrier-supportive signaling rather than inflammatory activation.

In parallel, dietary supplementation with capsiate-producing chili modified SCFA profiles over time. The reductions observed in acetic and propionic acids may reflect alterations in epithelial uptake or host–microbial metabolic interactions. However, fecal SCFA concentrations represent a composite of production, absorption, and utilization processes, which limits direct functional interpretation [33]. In contrast, the preservation of butyric acid, the primary oxidative substrate for colonocytes, is consistent with maintenance of colonic epithelial metabolic homeostasis [34].

The intestinal and systemic changes observed in this study may also be relevant to the skeletal outcomes identified in chili-supplemented mice. Dietary supplementation with capsiate-producing chili pepper was associated with increased femoral length, growth plate expansion, and higher osteocyte number and size, consistent with enhanced bone growth under the experimental conditions evaluated. Although the mechanisms underlying these observations remain unclear, previous studies have shown that inflammatory and redox-related pathways can influence bone remodeling and longitudinal growth [35]. In this context, the reduced intestinal expression of *TLR4*, *IL-6*, and *TNF-α* observed in the present study may be compatible with a systemic environment potentially less permissive to inflammation-associated bone resorption. Likewise, modulation of redox-associated genes may reflect conditions that have previously been linked to preservation of osteoblast activity under reduced oxidative stress [35,36].

Among the pathways potentially involved, *TRPV1* may represent a relevant candidate linking intestinal and skeletal responses. Experimental studies have reported that sustained *TRPV1* activation can promote NF-κB-dependent inflammatory signaling and cytokine production, including *TNF-α* and *IL-6*, which are associated with RANKL-mediated osteoclastogenesis [37]. In the present study, reduced intestinal *TRPV1* expression following exposure to capsiate-containing chili may therefore be compatible with modulation of signaling pathways previously implicated in the regulation of bone remodeling [35,36]. However, no direct measurements of osteoclast activity, bone turnover markers, or bone-specific gene expression were performed, and therefore any mechanistic relationship between intestinal *TRPV1* signaling and skeletal outcomes remains hypothetical.

Previous in vivo evidence further suggests that the skeletal effects associated with *TRPV1* signaling may depend on the balance between receptor activation and desensitization [38]. For example, *TRPV1* antagonism or genetic deletion has been reported to reduce bone erosion in inflammatory models [39], whereas vanilloid compounds such as capsaicin have been shown to modulate inflammatory and prostaglandin-related pathways in osteoblasts [40]. Although these findings derive from different experimental contexts, they provide a biological framework that may help contextualize the skeletal changes observed in the present study. In this regard, the concurrent reduction in inflammatory signaling and *TRPV1* expression may represent a plausible, although unconfirmed, mechanism potentially associated with enhanced femoral growth in chili-supplemented mice.

The simultaneous modulation of intestinal parameters and skeletal outcomes may also suggest a potential interaction along the gut–bone axis. Nevertheless, this interpretation should be considered exploratory, since no direct analyses linking intestinal responses with bone metabolism were conducted. Additional studies incorporating bone turnover markers, histomorphometric analyses, and targeted mechanistic approaches will be necessary to determine whether a causal relationship exists between capsiate-producing chili supplementation and skeletal adaptations.

In summary, supplementation with capsiate-producing chili pepper was associated with changes in growth, intestinal transcriptional responses, and femoral development in mice under non-pathological conditions. Increased body weight and femoral growth occurred without detectable alterations in adiposity or intestinal morphology, indicating that the observed responses were not accompanied by overt metabolic or tissue disruption.

The findings further indicate that this chili accession, characterized by a distinctive capsinoid and phytochemical profile, may be associated with modulation of multiple physiological processes, including intestinal homeostasis and skeletal development. Although the present study does not establish causal or mechanistic relationships, the combined intestinal and skeletal responses observed provide a basis for future investigations into the biological effects of capsiate-producing peppers and related bioactive compounds. Further studies integrating molecular, microbiological, and bone-specific approaches will be necessary to clarify the pathways involved and determine their physiological relevance.

## 4. Materials and Methods

### 4.1. Plant Material

Capsiate-producing chili seeds (*Capsicum annuum* L., accession 509-45-1, USDA/ARS/PGRU, USA) were kindly provided by Dr. Robert L. Jarret. Plants were cultivated at the Autonomous University of Querétaro, Amazcala campus (Querétaro, Mexico). Seeds were germinated in 200-cavity trays using peat moss as substrate under controlled conditions (22 ± 2 °C, 55 ± 5% relative humidity). When seedlings developed eight true leaves (≈2 months after emergence), they were transplanted into grow bags containing grit sand substrate. Forty days post-anthesis, plants were elicited with a single foliar application of 200 mmol hydrogen peroxide [17]. Fruits were harvested after elicitation, dehydrated in a universal oven at 40 °C for 84 h, ground in a pulverizer, and stored in the dark until use.

### 4.2. Characterization of Capsiate-Producing Chili (C. annuum L.)

Capsiate, capsaicin, and dihydrocapsaicin standards were obtained from Sigma-Aldrich (Buchs, Switzerland). Extraction of capsinoids was carried out following Fayos et al. (2019) [41] with minor modifications. Briefly, 200 mg of dried chili sample was mixed with 10 mL of HPLC-grade acetonitrile in 50 mL Falcon tubes (triplicate). Samples were sonicated for 3 h at 35 °C with agitation every 30 min and centrifuged (8000 rpm, 10 min, 4 °C), and the supernatants were filtered (0.45 μm). Filtrates were stored in amber vials at −20 °C until analysis. Quantification was performed using an Alliance 2695 HPLC (Waters Corporation, Milford, MA, USA) with a Zorbax Eclipse Plus C18 column (5 μm) (Agilent Technologies, Santa Clara, CA, USA), under isocratic conditions with acetonitrile:1% acetic acid in water (40:60, *v*/*v*) at 1.0 mL/min.

#### 4.2.1. Total Phenol and Flavonoid Quantification

Phenolic compounds were extracted from 200 mg of dried chili samples in 10 mL of methanol (triplicate). Flasks were covered with aluminum foil, sonicated at 25 °C for 30 min, and centrifuged at 5000 rpm for 10 min at 4 °C [42]. The supernatants were stored in the dark at 4 °C until analysis. Total phenols were determined using the Folin–Ciocalteu method [43] and expressed as mg gallic acid equivalents per g dry matter (mg GAE/g). Flavonoids were measured spectrophotometrically [44] and reported as mg rutin equivalents per g dry sample (mg RE/g).

#### 4.2.2. Antioxidant Activity Assessment

Antioxidant activity of *C. annuum* extracts was measured spectrophotometrically. DPPH RSA was performed following Feregrino-Pérez et al. (2011) [45] using 20 μL of extract and 200 μL of DPPH solution in 96-well plates, with absorbance recorded at 520 nm. Percent inhibition was calculated as % inhibition = [(Ac − As)/Ac] × 100, where Ac is the control absorbance and As is the sample absorbance. ABTS RSA was determined using 20 μL of extract and 230 μL of ABTS solution, with absorbance measured at 730 nm and results expressed as percent inhibition.

#### 4.2.3. Protein Determination

*C. annuum* chili powder was defatted by Soxhlet extraction with hexane [46] and digested in a microwave with 6 N HCl [47]. Digests were neutralized with 4 N NaOH and centrifuged at 5500 rpm for 20 min, and the supernatants were stored at 4 °C until analysis. Protein content was determined using the Bradford method [48] with Bradford reagent (Sigma-Aldrich).

#### 4.2.4. Determination of Soluble and Insoluble Fiber

Insoluble fiber and soluble fiber contents were determined by enzymatic–gravimetric methods using amylase, protease, and amyloglucosidase, according to the AOAC method [49].

### 4.3. Animals and Experimental Design

Twenty-one 21-day-old male C57BL/6J mice were used as a bioindicator model to assess the safety and potential functional effects of capsiate for agroalimentary applications. Animals were housed in the bioterium of the Instituto Nacional de Ciencias Médicas y Nutrición Salvador Zubirán (INCMNSZ) under standard conditions (21 ± 2 °C, 40–70% RH, 12 h light/12 h dark) with ad libitum access to food and water. Mice were randomly assigned to three groups (*n* = 7)—SD (standard chow), C30 (SD + 30 mg capsiate/kg feed), and C50 (SD + 50 mg capsiate/kg feed)—with 3–4 mice per cage. The experiment lasted from weaning (21 days) until 111 days of age, with body weight and feed intake recorded daily at a consistent time.

### 4.4. Growth Performance and Metabolic Characterization of Chili-Fed Mice

#### 4.4.1. Feed Intake, Capsiate Intake and Feed Conversion Ratio (FCR)

Weekly feed intake (FI) per mouse was recorded, and capsiate intake (CI) was estimated using the formula CI = (DI × W)/CC, where DI is daily intake, W is average body weight, and CC is capsiate content per kg of feed. Relative intake (RI) was calculated as the quotient of average weekly feed consumption and average weekly body weight. Feed conversion ratio (FCR) was determined as FCR = g feed/g weight gain, based on weekly weight gain and feed consumption.

#### 4.4.2. Body Composition Analysis by MRI

Body composition was measured following Castro-Rodríguez et al. (2020) [50] with modifications for mice. Each mouse was placed in a Perspex tube with a counterweight and scanned using a 2 MHz Whole-Body Composition Analyzer (Echo Medical Systems, Houston, TX, USA). A 30 g canola oil sample was scanned prior to the procedure for equipment calibration.

#### 4.4.3. Sample Collection and Metabolic Analysis

After 3 months of treatment, mice were fasted for 6 h, anesthetized with isoflurane, and euthanized. Blood was collected into EDTA tubes and centrifuged (3200 rpm, 15 min, 4 °C), and serum was stored at −80 °C. Triglycerides and cholesterol were measured enzymatically using a SynchronCX autoanalyzer (Beckman Coulter, Fullerton, CA, USA) [51]. Two-centimeter sections of the jejunum and colon, the liver, and various fat depots (mediastinal, retroperitoneal, omental, mesenteric, parametrial, epididymal) were collected, weighed, and used to calculate organ indices (% of body weight). Femurs were sectioned for length measurements and stored at −80 °C for further analyses, with additional samples fixed in 4% paraformaldehyde for histology.

### 4.5. Histology of Liver, Jejunum and Femur

Liver and jejunum samples were paraffin-embedded, sectioned (3–4 μm), mounted on positively charged slides, deparaffinized, and stained with hematoxylin–eosin (HE). Goblet cells were visualized with periodic acid–Schiff (PAS), and villus height, width, and crypt depth were measured using ImageJ 1.53v software (National Institutes of Health, Bethesda, MD, USA) [50]. Femurs were decalcified in 10% EDTA (pH 7.4) for 21 days, sectioned at 5 μm, and HE-stained [52]. Growth line area and osteocyte number were determined via semi-automated image analysis using Fiji/ImageJ as described by Miura et al. (2020) [53].

### 4.6. Gene Expression Analysis by RT-qPCR

Total RNA was isolated from the frozen jejunum, colon, and femur using TRIzol Reagent (Invitrogen^TM^, Thermo Fisher Scientific, Waltham, MA, USA). cDNA was synthesized by reverse transcription of DNase-treated RNA (1–3 μg) with the Transcriptor First-Strand cDNA Synthesis Kit (Roche Diagnostics, Mannheim, Germany). Quantitative PCR (qPCR) was performed on a LightCycler 2.0 instrument Roche Diagnostics, Mannheim, Germany) using Roche master mix and hydrolysis probes (Universal Probe Library, Roche) following standard protocols. Primer sequences used in this study are listed in Table 3. Briefly, Taq DNA polymerase activation and denaturation were carried out at 95 °C for 10 min, followed by 45 amplification cycles of 10 s at 95 °C, 30 s at 60 °C, and 1 s at 72 °C. All genes were analyzed under the same qPCR conditions and normalized to the housekeeping gene *Rpl32*, and relative expression levels were calculated using the ΔΔCT method [54].

### 4.7. Fecal SCFA Analysis

Feces were collected from mice at three time points: (1) one week (T1), (2) eight weeks (T2), and (3) twelve weeks (T3) of the experiment. SCFAs, including acetate, propionate, and butyrate, were quantified following Castro-Rodríguez et al. (2020) [50]. Briefly, fecal samples were homogenized, and the resulting supernatants were analyzed using a modified Folch method on a Varian 3380CX gas chromatograph (Varian, Palo Alto, CA, USA) equipped with a flame ionization detector and automatic split injection via a CP8400 autosampler (Varian, Palo Alto, CA, USA). Quantification was performed based on external calibration curves using SCFA standards.

### 4.8. Statistical Analysis

Statistical analyses were performed using GraphPad Prism 9. Data with normal distribution were analyzed by one-way ANOVA followed by Tukey’s post hoc test (α = 0.05), while non-normally distributed data were analyzed using the Kruskal–Wallis test (α = 0.05). Results are presented as the mean ± SEM.

## 5. Conclusions

Dietary exposure to a capsiate-producing chili pepper (*Capsicum annuum* L. accession 509-45-1) promoted somatic growth in mice, increasing body weight and longitudinal femoral development without detectable alterations in adiposity, intestinal morphology, or overall tissue integrity. To our knowledge, this is the first study demonstrating growth-promoting effects associated with dietary supplementation of a naturally capsiate-producing chili accession under non-pathological conditions.

The observed growth phenotype occurred alongside modulation of intestinal transcriptional markers related to sensory signaling, inflammatory pathways, redox-associated responses, and epithelial barrier function, suggesting that intestinal functional adaptations may contribute to the physiological responses associated with growth. In parallel, the preservation of body composition and intestinal structural integrity supports the notion that these responses occurred without evidence of overt metabolic or tissue disruption.

Although the present study does not establish direct mechanistic relationships between intestinal signaling and skeletal development, the combined phenotypic, molecular, and tissue-level findings provide a biological framework for future investigation of gut-mediated pathways involved in growth regulation. Overall, these results support the potential relevance of capsiate-producing chili peppers as phytogenic dietary components capable of promoting growth-related physiological responses, highlighting their possible value as natural alternatives to conventional growth-promoting strategies in animal production systems.

## Figures and Tables

**Table 1:** Characterization of capsiate-producing chili 509-45-1 (*Capsicum annuum* L.).

Composition of Chili	Content
Capsaicin (mg/g)	ND
Dihydrocapsaicin (mg/g)	ND
Capsiate (mg/g)	4.13 ± 0.17
Total Phenols (mg GAE/g)	34.99 ± 2.02
Total Flavonoids (mg RE/g)	27.37 ± 1.55
%RSA DPPH	16.11 ± 1.25
%RSA ABTS	62.48 ± 1.08
Protein (mg/g)	2.70 ± 1.47
Soluble Fiber (mg/g)	12 ± 0.00
Insoluble Fiber (mg/g)	356.16 ± 0.02

ND: not detectable. Data are expressed as means ± SEM. *n* = 3.

**Table 2:** Effect of capsiate-producing chili on body weight, organ index and fat distribution in mice.

Parameter Indicator	SD	C30	C50
Liver relative weight (%)	4.91 ± 0.36	5.29 ± 0.43	4.81 ± 0.43
Retroperitoneal fat (%)	0.31 ± 0.17	0.38 ± 0.12	0.40 ± 0.19
Sternum fat (%)	0.02 ± 0.01	0.02 ± 0.01	0.02 ± 0.01
Pancreatic fat (%)	0.22 ± 0.05	0.29 ± 0.09	0.20 ± 0.13
Mesenteric fat (%)	0.48 ± 0.14	0.49 ± 0.10	0.42 ± 0.15
Gonadal fat (%)	1.18 ± 0.22	1.44 ± 0.22	1.36 ± 0.49

Data are expressed as means ± SEM; *p* < 0.05.

**Table 3:** List of primers used in reverse transcription RT- qPCR.

Gene	Forward Sequence (5′-3′)	Reverse Sequence (5′-3′)
*SOD*	CAGGACCTCATTTTAATCCTCAC	TGCCCAGGTCTCCAACAT
*CAT*	CCTTCAAGTTGGTTAATGCAGA	CAAGTTTTTGATGCCCTGGT
*NRF2*	CACAGGGAGGACTTTGTGAGT	CAACAGTATTTCTGCCGCTGT
*IL6*	GCTACCAAACTGGATATAATCAGGA	CCAGGTAGCTATGGTACTCCAGAA
*IL10*	AGTTGACGGACCCCAAAAG	AGCTGGATGCTCTCATCAGG
*TNFα*	GACGGTGAGAGCCAGAGG	TGGTGGAAGTGATCACGAGT
*TRPV1*	CCTGTCCTGCATTGACACCT	GTTGGGGGTCTCACTGCTAC
*TLR2*	GGGCACCTACGAGCAAGAT	CTGCACTGGTGTCTGGAGTC
*TLR4*	CTGATCCATGCATTGGTAGGT	GGACTCTGATCATGGCACTG
*CD14*	AAAGAAACTGAAGCCTTTCTCG	AGCAACAAGCCAAGCACAC
*TJP1*	TGGGCCTAAGTATCCCGTCT	TGTGGATTTACCCGTCAGCC
*Claudin1*	GGAGCACCTTATCCCCGTTT	ACAGGAGGGAAGGCTTTTGC
*Rpl32*	ACGATCTTGGGCTTCACCAG	AGGTGGCTGCCATCTGTTTT

## Data Availability

The data supporting the findings of this study are available from the corresponding author upon reasonable request.

## Abbreviations

The following abbreviations are used in this manuscript:

ABTS	2,2′-azino-bis(3-ethylbenzothiazoline-6-sulfonic acid)
ANOVA	Analysis of variance
AOAC	Association of Official Analytical Chemists
CAT	Catalase
CD14	Cluster of differentiation 14
CI	Capsiate intake
C30	Diet containing 30 mg/kg capsiate
C50	Diet containing 50 mg/kg capsiate
DPPH	2,2-diphenyl-1-picrylhydrazyl
DNA	Deoxyribonucleic acid
EDTA	Ethylenediaminetetraacetic acid
FCR	Feed conversion ratio
FI	Feed intake
GAE	Gallic acid equivalents
H&E	Hematoxylin and eosin
HCl	Hydrochloric acid
HPLC	High-performance liquid chromatography
IL-6	Interleukin-6
IL-10	Interleukin-10
mRNA	Messenger ribonucleic acid
MRI	Magnetic resonance imaging
NaOH	Sodium hydroxide
ND	Not detectable
NRF2	Nuclear factor erythroid 2-related factor 2
PAS	Periodic acid–Schiff
qPCR	Quantitative polymerase chain reaction
RE	Rutin equivalents
RI	Relative intake
RNA	Ribonucleic acid
RT-qPCR	Reverse transcription quantitative polymerase chain reaction
RSA	Radical scavenging activity
SCFAs	Short-chain fatty acids
SD	Standard diet
SEM	Standard error of the mean
SOD	Superoxide dismutase
TJP1	Tight junction protein 1
TLR2	Toll-like receptor 2
TLR4	Toll-like receptor 4
TNF-α	Tumor necrosis factor alpha
TRPV1	Transient receptor potential vanilloid 1
USDA/ARS/PGRU	United States Department of Agriculture/Agricultural Research Service/Plant Genetic Resources Unit
